# Reference Intervals for Venous Blood Gas in Healthy Adults: A One-Year Prospective Study

**DOI:** 10.7759/cureus.74223

**Published:** 2024-11-22

**Authors:** Ayesha Akhtar, Syed Ali Abbas, Fatima Zaina, Gohar Fatima, Ahmed Wahab, Jamil Muqtadir

**Affiliations:** 1 Chest Medicine, Ziauddin University, Karachi, PAK; 2 Pulmonology and Critical Care, Ziauddin University, Karachi, PAK; 3 Pulmonology, Ziauddin University, Karachi, PAK; 4 Pulmonology and Critical Care, Liaquat National Hospital, Karachi, PAK; 5 Medicine, Ziauddin University, Karachi, PAK; 6 Infectious Diseases, Ziauddin University, Karachi, PAK; 7 Infectious Diseases, Dr. Ziauddin Hospital, Karachi, PAK

**Keywords:** arterial blood gas, blood gas analysis, metabolic physiology, reference intervals, venous blood gas

## Abstract

Background

Venous blood gas (VBG) investigation is a widespread option for arterial blood gas analysis because it is easier to draw and has a lower risk of complications during phlebotomy. This study aimed to establish reference intervals for the accurate analysis of VBG results as there is a lack of published data.

Method

Dr. Ziauddin Hospital hosted the study, which involved 182 healthy adults (96 female participants) between the ages of 20 and 76 years. They comprised 118 medical students (aged 20-32 years) and 64 volunteers (aged 20-76 years), the latter of whom were enrolled following the fulfillment of a health questionnaire. To avoid coagulation, a pre-heparinized syringe was used to draw venous blood from the dorsal or cubital vein (peripheral). The samples were sent directly to the laboratory, where the EasyBloodGasTM analyzer (Medica Corporation, Bedford, MA, USA) completed the analysis of the blood samples in 30 min.

Results

pH, pO2 (partial pressure of oxygen), pCO2 (partial pressure of carbon dioxide), bicarbonate, and electrolytes i.e. sodium (Na^+^), potassium (K^+^), chloride (Cl^-^), and calcium (Ca^+2^) were among the parameters included in non-parametric statistical analysis. The results established VBG reference intervals as follows: pH (7.29-7.43), partial pressure of oxygen (25-70 mmHg), partial pressure of carbon dioxide (35-59 mmHg), bicarbonate (22-30 mmol/L), Na^+^ (134-144 mmol/L), K^+^ (3.1- 4.6mmol/L), Cl^-^ (101-110 mmol/L) and ionized calcium (1.12-1.30 mmol/L).

Conclusion

This prospective study determined reference ranges for VBG measurements in healthy persons over one year, addressing the lack of published data from Pakistani institutions. Our research offers significant insights into the standard ranges for pH, pO2, pCO2, bicarbonate, electrolytes, and ionized calcium. The reference intervals established in this study can function as a dependable standard for doctors to appropriately interpret VBG data.

## Introduction

Blood gases provide a plethora of information regarding the body's acid-base balance, ventilation, and oxygenation. The term "blood gas analysis" in the medical field typically refers to the measurement of pH, lactate, partial pressure of carbon dioxide (pCO2), bicarbonate (HCO3-), and base excess (BE). To diagnose, treat, and monitor a variety of conditions, blood gas analysis provides vital and timely health information regarding the respiratory and metabolic processes of a patient [1].

Blood gas analysis was first used to measure the partial pressures of carbon dioxide and oxygen, and its application has been documented since the early 1900s. Subsequent blood gas analysis devices could measure the levels of glucose, lactate, creatinine, and electrolytes like sodium, potassium, chloride, ionized calcium, and magnesium in addition to hemoglobin quantitation and co-oximetry [2].

When assessing the blood gas status and acid-base balance of patients on mechanical ventilation, arterial blood gas (ABG) analysis is crucial. The ABG test has certain drawbacks, despite being very effective in assessing how well patients respond to treatment plans. Pain, arterial damage, infection, thrombosis with distal digit ischemia, bleeding, and aneurysm formation are the most frequent side effects of arterial punctures. Venous blood gas (VBG) sampling is a substitute for ABG sampling because it is a simple and easy process for obtaining venous blood [3].

As an alternative, VBG analysis has been proposed and is gaining popularity, particularly in emergency-care settings. Compared to ABG collection, VBG sampling is easier to execute and requires less staff training. Additionally, patients experience less pain, and there are fewer risks and complications for both the patient and the collector [1].

Three different venous samples can be used for VBG: mixed, peripheral, and central. Because of the strong correlation between central VBG, as determined by both clinical experience and research, central VBG was chosen. Peripheral VBG has been investigated as a backup for the central venous system [4]. Mixed VBG are a reasonable substitute for venous access in patients with pulmonary artery catheters (PACs). However, PACs should not be inserted alone for venous blood sampling.

Reference intervals (RIs) are tools used by clinicians to determine whether a patient is in a diseased or healthy clinical state. There are frequently good clinical and scientific justifications for variations in the RIs. As we move toward a single electronic health record and a national e-health framework, it is becoming increasingly important to provide physicians with results that enable accurate trustworthy clinical interpretation [5].

Data are used to create an RI that, at a given confidence level, represents 95% of the population. The Clinical and Laboratory Standards Institute advises that nonparametric ranking methods requiring a minimum of 120 individuals should be used to establish RIs for nonparametric datasets [6]; for parametric datasets without a minimum number requirement, the parametric method is appropriate. The 90% confidence intervals (CIs) for the RI limits must be determined for both approaches [6]. 

Good laboratory medicine practice requires a continuous review of RIs because the clinical significance of any individual's test result depends on the level of accuracy of the RI used for its comparison (interpretation). Few studies have validated the health-associated RIs that are currently used to interpret patient blood gas results [7]. This study was designed to develop RIs for the accurate analysis of VBG results at Dr. Ziauddin Hospital because of the lack of published data from Pakistani hospitals.

## Materials and methods

This comparative study was conducted from January 2023 to December 2023 at Dr. Ziauddin Hospital in Karachi, Pakistan.

Selection of reference individuals

In choosing the RIs, a total of 182 healthy adults (96 of whom were female) between the ages of 20 and 75 years were included. Among them were 118 medical students, between the ages of 20 and 32, and 64 volunteers, between the ages of 20 and 75, who were recruited after answering a health questionnaire. Participants included adults in good health or whose conditions were unlikely to impact blood gases and acid-base equilibrium [8]. The inclusion norms were not smoking, having no history of respiratory diseases, or anorexia nervosa diagnoses or treatments within the previous few months, and no history of vomiting or diarrhea within the previous week. Vulnerable populations, including pregnant women and seniors over 75, were not asked to participate because samples were required following an overnight fast. Using a health questionnaire that recorded the weight and height of the study participants, compliance with the criteria mentioned above was determined. All the participants provided informed consent [1].

Ethical approval

Dr. Ziauddin Hospital in Karachi, Pakistan's Ethical Review Committee, provided ethical approval (reference number: 4981275AAPUL) before starting the study.

Sampling

Participants required the previously stated anthropometric measurements, informed approval, health inquiry form, and donation of a small sample of blood [9]. Patients' verbal informed consent was obtained, and pertinent information such as age, sex, and length of hemodialysis was recorded on proformas [8]. To avoid coagulation, a pre-heparinized syringe was used to draw venous blood from the dorsal or cubital veins (peripheral) [4]. Samples were collected with minimum delay (less than 10 min) [10].

Statistical analysis and data collection

Samples were then sent directly to the lab, where they were examined using the EasyBloodGasTM analyzer (Medica Corporation, Bedford, MA) in less than 30 min [8]. After entering all data, GraphPad Prism Statistics for Windows, Version 10.201 (GraphPad Software, San Diego, CA) was used for analysis. For quantitative data, the means and standard deviations were computed [9]. The International Federation of Clinical Chemistry and Laboratory Medicine rules were followed for the non-parametric calculation of RIs [11]. The Grubbs (alpha = 0.05) test was used to screen for outliers [12]. The Mann-Whitney U test was used to investigate differences based on sex [13]. Analysis of variance or Welch’s test was used to evaluate age-dependent differences [14]. The information is displayed as the 90th percentile CIs around the 2.5-97.5 percentile ranges.

## Results

Venous blood gas RI

For the critical care parameters, the RIs were computed using a non-parametric method. A total of 200 healthy adults volunteered to take part in the study, and 18 of them said they had used tobacco products such as cigarettes, cigars, or e-cigarettes (electronic cigarettes) 12 months prior. Following the exclusion of these subjects, 182 (86 men (47.25%) and 96 women (52.7%) with mean ± SD and ages of 45.44 ± 17.66 years) had their VBG results included in the analysis used to determine the VBG RI. Table 1 shows the numbers of men and women in each age group.

**Table 1:** Age bands of men and women

Age	Men	Women	Total
18-25	21	18	43
26-35	46	36	83
36-45	7	14	17
46-55	7	12	20
56-65	4	12	15
66-75	1	4	4
Total	86	96	182

pH, bicarbonate (HCO3-), electrolytes (Na+, Cl-, K+), partial pressure of carbon dioxide (PCO2), partial pressure of oxygen (O2), and ionized calcium were observed, as shown in Table 2.

**Table 2:** Reference intervals for VBG in healthy adults (n=182) pO2, partial pressure of oxygen; pCO2, partial pressure of carbon dioxide; mmHg, millimeter of mercury; mmol/L, milli moles per liter; VBG, venous blood gas.

Measured	Reference Interval (2.5-97.5 percentile)	90% Confidence Interval Lower Reference Limit	90% Confidence Interval Upper Reference Limit
pH	7.29-7.43	7.28-7.33	7.42-7.43
pO2 (mmHg)	25-70	23-31	53-78
pCO2 (mmHg)	35-59	33-36	51-68
Bicarbonate (mmol/L)	22-30	22-23	28-30
Sodium (mmol/L)	134-144	133-137	142-146
Potassium (mmol/L)	3.1-4.6	3-3.2	4.3-4.7
Chloride (mmol/L)	101-110	101-102	108-111
Ionized calcium (mmol/L)	1.12-1.30	1.07-1.16	1.26-1.3

Result analysis

For both pO2 and pCO2, a single outlier was identified and eliminated using the Grubbs test (alpha = 0.05). The analysis contained all findings for each analyte. Sex-dependent differences were statistically significant for pCO2, pO2, and sodium levels. Men had lower venous pCO2 (33-61 mmHg) than women (34-68 mmHg). Similar results were observed for sodium and pO2, with values of 133-144 mmol/L in men and 134-146 mmol/L PO2 in women and 23-71 mmHg in men and 25-78 mmHg in women, respectively. Despite this, the merged reference limits were considered suitable because the CIs for the higher and lower reference limits for male and female subjects encompassed those for the merged reference limits. The differences were negligible in every other instance and a combined RI was deemed appropriate.

**Figure 1:**
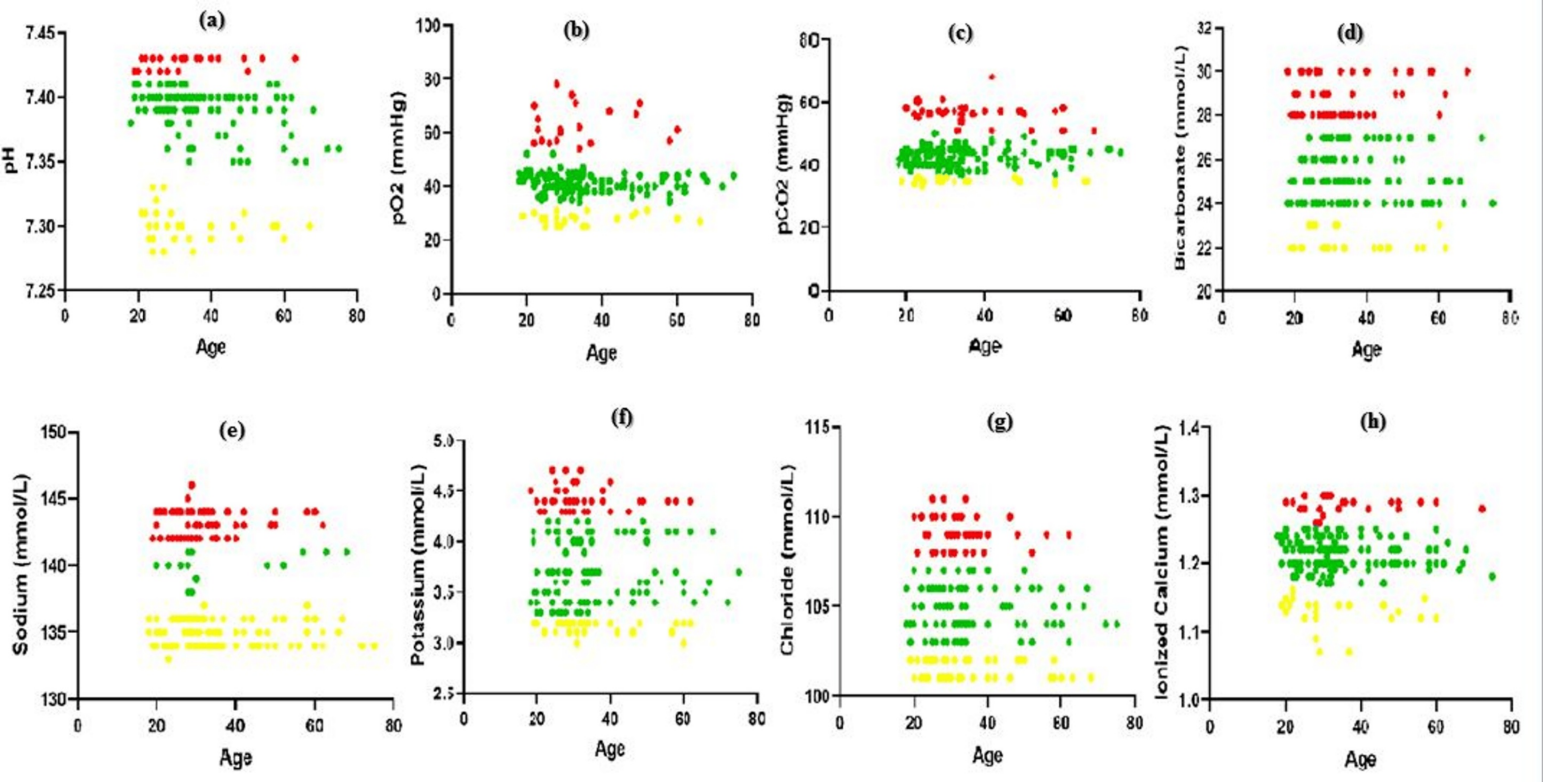
Graphical representation of the results for VBG RI in adults; lower, upper and median are represented in yellow, red and green for (a) pH, (b) pO2, (c) pCO2, (d) bicarbonate, (e) sodium, (f) potassium, (g) chloride, and (h) ionized calcium. pO2, partial pressure of oxygen; pCO2, partial pressure of carbon dioxide; mmHg, millimeter of mercury; mmol/L, millimol per liter; VBG, venous blood gas; RI, reference interval.

Participant’s number was not evenly scattered across age groups, as shown in Table 1. The age bands were split into two groups (18-35 years) and (36-75 years) before the age-dependent variances were evaluated. Age-dependent differences were statistically significant only for sodium. Once again, the differences were negligible, and when paired with the ambiguity of the divided RIs, a combined RI was deemed suitable in every situation. The VBG RI found in our reference population is displayed in Table 2 and Figure 1.

## Discussion

Clinical medicine, as well as cardiorespiratory and metabolic physiology, has been revolutionized by blood gas analysis [15]. Measured pH and the derived acid-base variables can not only aid in diagnosis, but also in determining the severity of a specific condition (metabolic acidosis in sepsis), the level of monitoring needed, and the appropriate course of further intervention [16]. Arterial blood was typically used to measure these variables. It can be difficult to obtain arterial blood, especially in the early stages of resuscitation, when arterial line access needs to be established [17].

This study aimed to establish RIs for adult peripheral venous samples for pH, bicarbonate, base excess, and other values.

Ress et al. conducted a prospective study to determine the VBG RI in adults by comparing the theoretical VBG RI data from their meta-analysis with the measured VBG values [1]. The measured and theoretical RIs from Ress's modeling were generally comparable; our study showed 7.29-7.43 pH, while the modeling estimated 7.30-7.43 VBG RI. The quantitative upper and lower reference limits were in line with earlier reports in the literature and textbooks. In particular, the reference range for venous pH has long been established as 7.31-7.41, or a slightly different range [9]. Our findings support this range of healthy adults, indicating that historical ranges can still be used to interpret pediatric test results (Figure 1a).

According to Klaestrup et al., women typically have a higher respiratory rate than men, which may account for their lower arterial pCO2 and higher pH [7]. Women had lower venous pCO2 but no change in pH, according to Ress et al. We also discovered a sex difference in pCO2, with a higher value for men, but not for pH in our investigation. Ress et al. stated that there was no discernible age- or sex-dependent difference in pO2. However, a difference was observed for female venous pO2 (25-78 mmHg) and male venous pO2 (23-71 mmHg) in our study. However, age- and sex-specific RIs were deemed unnecessary due to the small and clinically insignificant nature of these changes. Figures 1b, 1c show plots of adult VBG RI values for pO2 and pCO2, respectively.

Ress et al. discovered significant variations in hypothetical RI limits of pO2. According to a systematic review, VBG pO2 is an unpredictable measure of respiratory failure. Every meta-analysis that calculated variations in this parameter between ABG and VBG revealed substantial variance and potential publication bias [1,9]. Therefore, the RI values predicted in this study may also be unreliable.

Bicarbonate (Figure 1d) showed no statistically significant variations based on age or sex, indicating that the RI was fairly comparable. Overall, the results were similar between estimation of a VBG RI of Na+ 135 to 143 mmol/L, K+ 3.6 to 4.5 mmol/L, Cl- 101 to 110 mmol/L, Ca+2 1.14 to 1.29 mmol/L by Ress et al. and the present VBG RI study, which calculated RI of 134 to144 mmol/L, of 3.1 to 4.6 mmol/L, 101 to 110 mmol/L, and 1.12 to 1.30 mmol/L, respectively (Figures 1e-1h). Although there were statistically significant variations in sodium data based on age and sex, The Canadian Health Measures Survey measured the serum concentrations of Na+, K+, and Cl- in thousands of healthy Canadians aged 3 to 79 years and found statistically significant age and sex-dependent differences in the sodium and chloride data. Age and sex-specific differences were found for sodium in this study, but lower and higher reference limits were comparable across partitions, and the large sample size most likely contributed to the statistical significance that was found [18].

According to our data age and sex-dependent differences were statistically significant; however, the dissimilarities were minor, and the calculated RI was significantly uncertain. The non-uniform dispersal of different age groups in the reference population and the lower male-to-female ratio in our study population were weaknesses that hindered our ability to compute trustworthy age or sex-specific RIs.

The reference population (RI) for venous blood gas samples analyzed on the EasyBloodGasTM analyzer in Pakistan's metropolitan area was the adult population determined in this study. The accurate elucidation of critical care test results in laboratories that are becoming increasingly useful in clinical settings will be made easier through this study. As advised by the CLSI EP28-A3c guidelines, it is crucial that these reference standards are validated for the analytical platform of each laboratory, intended sample matrices, and local pediatric population prior to implementation.

## Conclusions

This prospective study addressed the lack of published data from Pakistani hospitals by establishing reference ranges for VBG values in healthy people. The typical ranges for pH, pO2, pCO2, bicarbonate, electrolytes, and ionized calcium are further elucidated by our findings, which give useful insights into these parameters. The RIs that were developed as a consequence of this study have the potential to serve as a trustworthy standard for doctors to use when appropriately interpreting VBG data. This work makes a contribution to the current body of research by presenting reference ranges for VBG values in healthy people that are both up to date and thorough, as well as insights on the relevance of VBG analysis in clinical settings. This study will assist improve diagnostic accuracy and clinical decision-making in critical care settings. It will also offer a basis for generating reference intervals that are particular to a population and bring attention to the necessity of continued research into the clinical uses of VBG parameters. This study highlights the need for creating reliable reference ranges for VBG measures in order to enhance the quality of treatment provided to patients and the results they experience.
